# Characteristics of idiopathic multicentric Castleman disease in Spain: results of the registry-based ARCANA multicenter study

**DOI:** 10.3389/fonc.2026.1963499

**Published:** 2026-10-19

**Authors:** Jose-Tomas Navarro, Santiago Montes-Moreno, Grisell Starita Fajardo, Manuel Espeso de Haro, Pedro Durán-del-Campo, Eva Gonzalez-Barca, Antonio Gutiérrez, Ana Jiménez-Ubieto, Alejandro Martín García-Sancho, Araceli Rubio-Martínez, Rosario Sánchez-Martínez, Paloma García-Martín, Pablo Lorente-Alegre, Iñigo Olazabal Eizaguirre, Angel Ramirez Payer, Guillermo Rodriguez, Antonio García-Guiñon, Andrés González-García

**Affiliations:** 1 Department of Hematology, Institut Català d’Oncologia. Josep Carreras Leukaemia Research Institute, Universitat Autònoma de Barcelona, Badalona, Spain; 2 Department of Anatomic Pathology, Valdecilla/IDIVAL, Universidad de Cantabria, Santander, Spain; 3 Department of Internal Medicine, Hospital Universitario Ramón y Cajal, Madrid, Spain; 4 Department Hematology and Hemotherapy, Hospital Regional Universitario de Málaga, Málaga, Spain; 5 Department of Internal Medicine, Hospital Universitario Puerta de Hierro Majadahonda, Majadahonda, Spain; 6 Department of Hematology, Institut Català D’Oncologia Hospitalet, IDIBELL, Universitat de Barcelona, L’Hospitalet de Llobregat, Spain; 7 Department of Hematology, Son Espases University Hospital, Palma, Spain; 8 Department Haematology, Hospital 12 de Octubre, Madrid, Spain; 9 Department Hematology, Hospital Universitario de Salamanca-IBSAL, CIC- IBMCC (USAL-CSIC), CIBERONC, Salamanca, Spain; 10 Department Hematology, University Hospital Miguel Servet, Zaragoza, Spain; 11 Internal Medicine Department, Dr. Balmis General University Hospital, Alicante Institute for Health and Biomedical Research (ISABIAL), Alicante, Spain; 12 Department of Hematology and Hemotherapy, San Cecilio University Clinical Hospital, Granada, Spain; 13 Department of Hematology, Hospital Arnau de Vilanova-Lliria, Valencia, Spain; 14 Department of Hematology, Hospital Universitario Donostia, Donostia, Spain; 15 Department of Hematology, Hospital Universitario Central de Asturias, Oviedo, Spain; 16 Department of Hematology, Hospital Universitario Virgen del Rocío, Sevilla, Spain; 17 Department of Hematology, Hospital Arnau de Vilanova, Lleida, Spain; 18 Systemic Autoinmmune Diseases Unit, Internal Medicine Department, Hospital Universitario Ramón y Cajal, IRYCIS, Madrid, Spain; 19 Faculty of Medicine and Health Sciences, Universidad de Alcalá (UAH), Madrid, Spain

**Keywords:** epidemiology, idiopathic multicentric Castleman disease, prevalence, response, treatment

## Abstract

**Objectives:**

We aimed to estimate the prevalence of iMCD in Spain by applying the current international consensus criteria.

**Methods:**

This was an observational, multicenter, cross-sectional study that was conducted in 20 Spanish hospitals. The diagnosis of iMCD and the histological subtype were confirmed by a centralized scientific committee by applying the international consensus criteria. The local clinical, laboratory, and pathology reports were reviewed.

**Results:**

Between February 2023 and April 2024, 30 patients with a documented diagnosis of iMCD were included in the study. The estimated prevalence of iMCD was 0·61 cases per 100,000 inhabitants (95% CI, 0·39–0·83); 23 subjects (76·7%) were classified as iMCD-NOS, and 7 (23·3%) were classified as iMCD-TAFRO. Twenty-two patients (73·3%) had received therapy for iMCD prior to their inclusion in the study. There are currently 21 patients (70%) on treatment, the most common being the use of siltuximab (19 patients; 90·4%). Complete response, partial response, and stable disease were reported in 11 (36·7%), 8 (26·7%), and 9 (30%) patients, respectively, with current treatment.

**Conclusion:**

The prevalence of clinical cases of iMCD in Spain comparable to that reported in the literature. Siltuximab is currently the most commonly used therapy. Most patients under treatment showed a favorable clinical response.

## 1 Introduction

Castleman disease (CD), first described in 1956 (1), represents a heterogeneous group of different disease entities that share a common morphological pattern in the lymph nodes (2, 3). Clinically, CD is classified into two main subtypes according to lymph node involvement: unicentric CD (UCD), a benign form with a single affected lymph node region, and multicentric CD (MCD), a potentially fatal variant with multiple affected lymph nodes and episodic systemic inflammatory symptoms (4–6). MCD can be further divided into the following subclasses: idiopathic MCD (IMCD), human herpes virus-8 (HHV8)-associated MCD (HHV8-MCD), polyneuropathy, organomegaly, endocrinopathy, monoclonal plasma cell disorder, and skin change (POEMS)-associated MCD (POEMS-MCD) (7, 8).

Idiopathic MCD, which accounts for 30–50% of all MCD cases (7), is characterized by systemic inflammation, generalized lymphadenopathy, polyclonal lymphoproliferation, and multiple organ system dysfunction (9, 10). Common clinical manifestations of iMCD include lymphadenopathy, fever, night sweats, fatigue, hepatosplenomegaly, and anemia (5, 9); however, the clinical presentation and severity of the disease vary greatly among patients (6, 9). IMCD is subclassified into two clinical subtypes: iMCD-TAFRO, characterized by thrombocytopenia, anasarca, fever/ascites, reticulin fibrosis/renal dysfunction, and organomegaly, with a more aggressive and life-threatening profile, and the iMCD-not-otherwise specified (iMCD-NOS) variant (7). Other variants within the iMCD spectrum have been previously described. The so-called oligocentric CD has recently been proposed as a less severe form with a limited number of affected lymph nodes and not fulfilling all iMCD criteria (11). Idiopathic plasmacytic lymphadenopathy (IPL) is a nonaggressive form characterized by thrombocytosis, hypergammaglobulinemia, and plasma cell-rich histopathological features (12).

Owing to its clinical heterogeneity and overlap with other conditions, as well as the lack of disease-specific biomarkers, the diagnosis of iMCD remains a significant challenge, and it is often made by the exclusion of similar clinical and pathological conditions (10, 13, 14). Accordingly, the publication of the Consensus Diagnostic Criteria for iMCD in 2017, issued by an international multidisciplinary expert panel, represents a significant step forward (7). The diagnostic criteria for TAFRO syndrome were first published in 2016 (15) and were updated in 2019 (16). Since then, further consensus initiatives toward improving the diagnosis of iMCD with an integrated multidisciplinary approach have been undertaken (14). A critical step following the diagnosis of iMCD was the publication of evidence-based consensus guidelines for the management of iMCD by an international expert panel from the Castleman Disease Collaborative Network (CDCN) (8).

There has been little research on the epidemiology of iMCD. Two US studies were published before the release of consensus diagnostic criteria (17, 18). A recently published U.S. study using a claims database, on the basis of the 2017 consensus criteria, estimated the 2018 annual prevalence and incidence of iMCD to be 9·7 per million and 3·1 per million, respectively (19). Similarly, an estimated annual incidence of 2·4–5·8 per million individuals and a prevalence of 4,180–14,900 cases of MCD in Japan have been reported but without specific data on iMCD (20). Importantly, no epidemiological study on iMCD has been conducted in Europe.

The present study aimed primarily to estimate the prevalence of iMCD, and the secondary objectives were to assess the clinical and pathological features of the enrolled patients and to determine their treatment patterns and clinical response in a real-world setting in Spain.

## 2 Materials and methods

### 2.1 Study design

This was an observational, multicenter, cross-sectional study conducted on patients diagnosed with iMCD. The cohort for this analysis consisted of cases of patients with a documented diagnosis of iMCD (HHV-8 negative/HIV negative) who were still alive and under monitoring at the beginning of the study according to the international, evidence-based consensus diagnostic criteria for iMCD (7)(**Supplementary Figure 1**). The study also included a prospective period to evaluate the incidence of this condition, the results of which are not yet available and will be published elsewhere.

Data from the patients included in this analysis were cross-sectionally collected from 20 Spanish hospitals for CD at the beginning of the study. The diagnosis of iMCD and its histologic subtype were confirmed by a centralized scientific committee that reviewed the local clinical, laboratory, and pathology reports from excisional lymph node biopsies, provided by the investigators at each participating institution upon the inclusion of patients. Similarly, CD types other than iMCD were discarded. The scientific committee was composed of an internist, a hematopathologist, and a hematologist.

The study protocol was reviewed and approved by the Ethics Committee of the Ramon y Cajal University Hospital (CodNr· 060/23; Madrid, Spain). Written informed consent to participate in the study was obtained from all patients before enrollment in the study.

### 2.2 Study population and data collection

Patients were eligible for the study if 1) they had a documented diagnosis of iMCD (HHV-8 negative/HIV negative) according to the 2017 International iMCD Consensus Diagnostic Criteria (7) and were subsequently confirmed by the scientific committee; 2) they were under current monitoring; and 3) they were older than 18 years of age.

### 2.3 Study endpoints and outcome measures

The primary endpoint was the estimation of disease prevalence. Only cases with a diagnosis consistent with iMCD (HHV-8 negative/HIV negative) and those under follow-up at the beginning of the study were considered for disease prevalence estimation.

Second, the following information was collected and described: sociodemographic characteristics (age, sex, and race); years after diagnosis (from the date on which a diagnosis compatible with iMCD was made); treatment for iMCD (previous therapies, with the treatments by lines of administration, and current treatment); iMCD clinical subtype (TAFRO/NOS); iMCD histological subtype (hypervascular/mixed/plasmacytic) according to local pathology report; disease severity; and iMCD response criteria at inclusion according to the Castleman Disease Collaborative Network (CDCN) (8) (no evidence of disease (complete response) versus evidence of disease (partial response, stable disease, or disease progression).

### 2.4 Statistical analysis

The sample size for this prevalence analysis was based on potential cases that could be included in the study from the 20 participating centers.

For the analysis of the primary objective, the prevalence was estimated as the total number of diagnosed iMCD cases under follow-up at the time of the site initiation visit (during the 24 h period on the day the site initiation visit occurred), with the reference adult population (i.e. ≥18 years) of the participating sites (4,908,959) used as the denominator. Patients who died prior to the initiation visit at a site were not considered cases of iMCD in the prevalence calculation. Prevalence was expressed as the number of cases per 100,000 inhabitants with a 95% confidence interval.

For the analysis of the secondary objectives, given the descriptive nature of the study, the mean, median, standard deviation, and interquartile range (IQR) were calculated for continuous variables, and the number and percentage were calculated for categorical variables, as appropriate. Missing values were not considered in the calculation of percentages.

All patients who met the selection criteria were included in the full analysis set (FAS) population. All analyses were performed using the statistical program Statistical Package for the Social Sciences (SPSS) v26.

## 3 Results

Between August 2023 and January 2024, 37 patients from 15 of the 20 participating centers were included in this study. Seven patients were excluded because they did not have a documented diagnosis of iMCD (HHV-8 negative/HIV negative), leading to the inclusion of 30 patients in the FAS population (**Figure 1**). Further information on the reasons for exclusion is provided in **Supplementary Table 1**. Notably, two patients had lymphadenopathies ranging from 2 to 4, were confined to the same anatomical region, and did not have the required minor criteria; hence, they were classified as having oligocentric CD (11). Patients were diagnosed with iMCD between September 1998 and June 2023; specifically, eight (26·7%) patients were diagnosed with iMCD before 2017, and 22 (73·3%) were diagnosed during or after 2017. The enrolled subjects had a median age of 48 years (IQR, 42–60 years); 50% were male, and 76·7% were Caucasian (**Table 1**). The median time since the diagnosis of iMCD was 47·4 months (IQR, 24·9–94·1).

**Figure 1:**
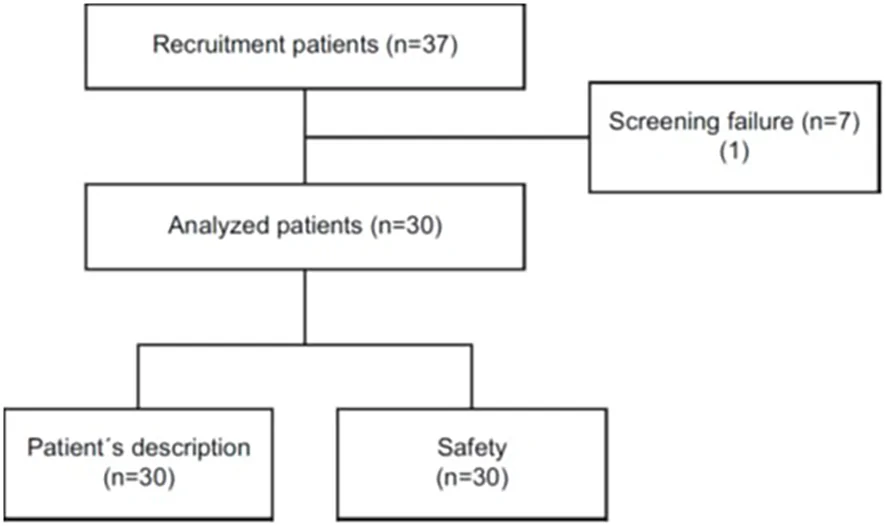
Patients’ disposition. (1) The list of screening failures is displayed in **Supplementary Table 1**.

**Table 1:** Demographic and clinicopathological characteristics (N = 30).

Characteristic	N (%)
Age, years, median (IQR)	48 (42–60)
Sex	
Female	15 (50)
Male	15 (50)
Ethnicity	
Caucasian	23 (76·7)
Other^a^	7 (23·3)
Time from diagnosis, months, median (IQR)^b^	47·4 (24·9–94·1)
iMCD subtype	
NOS	23 (76·7)
TAFRO	7 (23·3)
iMCD severity at diagnosis	
Non severe	21 (70)
Severe	9 (30)
iMCD histopathological subtype	
Plasmacytic	13 (43·3)
Mixed	10 (33·3)
Hypervascular	4 (13·3)
Not available	3 (10)
Response to treatment	
Complete response	11 (36·7)
Stable disease	9 (30)
Partial response	8 (26·7)
Progression disease	2 (6·7)

iMCD, idiopathic multicentric Castleman disease; IQR, interquartile range; NOS, not otherwise specified; TAFRO, thrombocytopenia, anasarca, fever, reticulin fibrosis, organomegaly.

^a^ Latin (3), Black (2), Asian (1), Arab (1).

^b^ Time from the date on which a diagnosis for iMCD was made to the informed consent date.

### 3.1 Prevalence of iMCD

The estimated prevalence of iMCD was 0·61 cases per 100,000 inhabitants (95% CI, 0·39–0·83).

### 3.2 Clinical features

Twenty-three subjects (76·7%) presented with a clinical profile of the iMCD-NOS subtype, and seven (23·3%) were classified as having iMCD-TAFRO. In addition, 9 (30%) patients were considered to have severe iMCD (**Table 1**).

Twenty-one patients presented with comorbidities at any time, of which 17 (81%) had more than one comorbidity, with autoimmune-autoinflammatory diseases (n=11) and hypertension (n= 7) being the most frequent. Eighteen patients (60%) had at least one comorbid condition before the diagnosis of IMCD (**Table 2**), 7 of whom (38·9%) presented more than one, and 13 patients (43·3%) developed comorbidities following iMCD diagnosis (**Table 2**), with 10 of them (76·9%) affected by more than one. At iMCD diagnosis, the most common comorbidities were autoimmune-autoinflammatory diseases (n=8), including hypothyroidism (n=3), and diabetes mellitus (n=3). Similarly, among the comorbidities and/or complications following iMCD diagnosis, hypertension was the most frequent (n=5), followed by hypothyroidism (n=2) (**Table 2**).

**Table 2:** Comorbidities diagnosed before and after iMCD diagnosis.

Comorbidities	Comorbidities presenting prior to iMCD diagnosis, n (%)^*^	Comorbidities presenting after iMCD diagnosis, n (%)^*^
Patients with any comorbidities	18 (60)^a^	13 (43·3)^a^
Autoimmune/autoinflammatory diseases	8 (44·5)^b^	3 (23·1)^c^
Hypothyroidism	3 (16·7)^b^	2 (15·4)^c^
Thrombotic thrombocytopenic purpura	1 (5·5)^b^	··
Psoriasis	1 (5·5)^b^	··
Graves’ disease	1 (5·5)^b^	··
Autoimmune hemolytic anemia	1 (5·5)^b^	··
Immune thrombopenia	1 (5·5)^b^	1 (7·7)^c^
Nonhaematological neoplasms	1 (5·5)^b^	1 (7·7)^c^
Squamous cervical intraepithelial neoplasia	1 (5·5)^b^	··
Benign prostatic hypertrophy	··	1 (7·7)^c^
Thrombotic event	··	1 (7·7)^c^
Thrombus in superior vena cava	··	1 (7·7)^c^
Other comorbidities	12 (66·7)^b^	12 (92·3)^c^
Diabetes Mellitus	3 (16·7)^b^	1 (7·7)^c^
Hypertension	2 (11·1)^b^	5 (38·5)^c^
Obesity	2 (11·1)^b^	··
Carpal tunnel syndrome	2 (11·1)^b^	··
Other less frequent comorbidities	11 (61·1)^b^	11 (84·6)^c^

iMCD, Idiopathic multicentric Castleman disease.

^*^ Patients may present with more than one comorbidity/complication.

^a^ Percentages were calculated relative to the total number of patients with iMCD included in the study (n = 30).

^b^ Percentages were calculated relative to the total number of patients with comorbidities prior to iMCD diagnosis (n = 18).

^c^ Percentages were calculated relative to the total number of patients with comorbidities after iMCD diagnosis (n = 13).

### 3.3 Histopathological features

Lymph node biopsies were performed for 30 patients. The plasmacytic histopathological subtype was the most prevalent (43%), followed by the mixed and hypervascular subtypes in 33·3% and 13·3% of the patients, respectively (**Table 1**). More specifically, plasmacytic was the most common subtype in the iMCD-TAFRO subgroup (57·1%), whereas plasmacytic (36·8%) and mixed (36·8%) subtypes were the most prevalent among iMCD-NOS patients.

Six individuals had undergone biopsies prior to the definitive diagnosis of IMCD, including 6 core needle biopsies and two excisional biopsies. Reactive lymphadenopathy and nondiagnostic CD features were reported in all previous biopsies.

### 3.4 Results of treatment and current status

The status of the patients at the inclusion in the study was complete response, stable disease, partial response, and progressive disease in 11 (36·7%), 9 (30%), 8 (26·7%), and 2 (6·7%) patients, respectively (**Table 1**).

Twenty-eight of 30 patients ever received treatment for iMCD. According to available information, two patients did not receive any treatment for iMCD. Twenty-one patients (70%) were receiving treatment for iMCD at the time of the data collection (current treatment), and twenty-two had received previous therapies for the disease. Furthermore, six patients out of the 21 were receiving current treatment as their first-ever therapy for the disease.

Among the 21 patients who were receiving current treatment, the most commonly prescribed therapy was anti-IL-6 mAb with/without corticosteroids (20, 95·2%; 19 with siltuximab (90·4%), with 18 on monotherapy and one on siltuximab combined with corticosteroids). Among the 9 patients who were not receiving treatment, 4 had been treated with anti-IL6 therapy, and the treatment was discontinued because of adverse events, but the patients had stable disease; 2 patients were treated with rituximab (one had a complete response, and the other had a partial response), and one additional patient received only corticosteroids and achieved a partial response. The remaining 2 patients had not been treated, and they had paucisymptomatic stable disease.

Twenty-two patients (73·3%) had received previous therapy for iMCD for a maximum of seven consecutive lines of treatment; however, most patients had received one (n=6 [27·3%]), two (n=6 [27·3%]), or three lines (n=4 [18·4%]) of treatment. The most frequent prior treatment patterns were anti-interleukin-6 monoclonal antibodies (anti-IL-6 mAb) with/without corticosteroids (13, 59·1%), rituximab with/without corticosteroids (10, 45·5%) and a chemotherapy-based regimen (10, 45·5%) (**Table 3**).

**Table 3:** Previous treatment patterns.

Treatment	N (%)^*^
Anti-IL-6 with/without corticosteroids	13 (59·1)
Rituximab with/without corticosteroids	10 (45·5)
Chemotherapy-based regimen	10 (45·5)
Corticosteroid monotherapy	6 (27·3)
Immunomodulator with/without corticosteroids	5 (22·7)
Other (radiotherapy)	1 (4·5)

Anti-IL-6, Anti-interleukin-6.

^*^ Percentages calculated over the total number of patients with previous treatment (N = 22). Patients may have received more than one line of treatment.

Regarding overall therapies (current plus prior treatments) by lines of treatment, the most frequent first- and second-line treatments were anti-IL-6 mAb with/without corticosteroids (46.4% and 38.9%, respectively), followed by rituximab with/without corticosteroids (17.9& and 27.8%, respectively). All the therapies by line of treatment are displayed in **Figure 2**; **Supplementary Table 2**.

**Figure 2:**
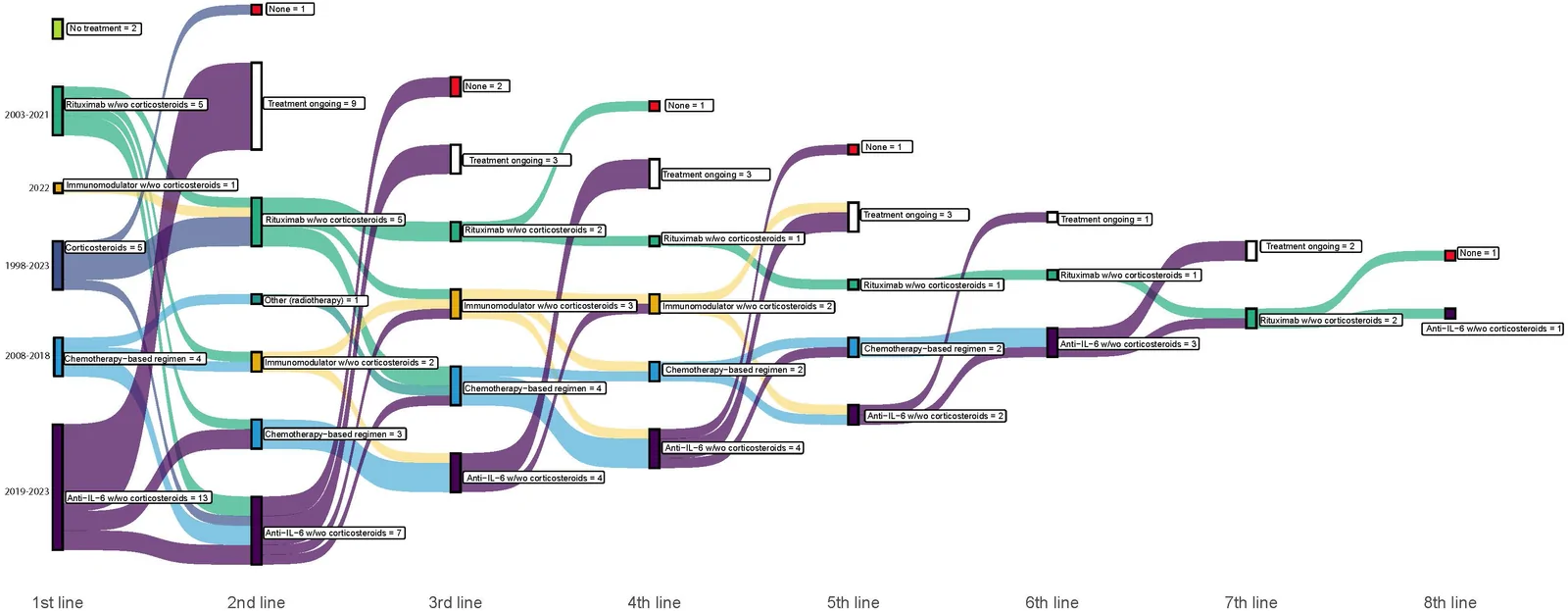
Sankey diagram of treatment patterns by line (previous and current). w/wo, with or without.

## 4 Discussion

The ARCANA study is the first study to evaluate the epidemiology of iMCD in Spain. Local pathology reports and clinical and laboratory reports from 30 patients belonging to 15 institutions were reviewed by a centralized scientific committee using the 2017 consensus diagnostic criteria for iMCD (7). The estimated prevalence of iMCD in our study was 0·61 cases per 100,000 inhabitants (equivalent to 6·1 cases per million inhabitants). These results are slightly lower than the prevalence estimated in a recently published retrospective health care claims data analysis performed in the U.S., in which the prevalence of iMCD was estimated to be 6·9 and 9·7 per million inhabitants in 2017 and 2018, respectively (19). However, unlike in our study, iMCD diagnoses were recorded based on the clinically modified International Classification of Diseases (ICD-10-CM) and Current Procedural Terminology codes, which are administrative coding systems. Furthermore, the fact that the diagnostic codes were not confirmed by pathology reports could have limited the certainty of iMCD diagnoses according to the authors of that report (19). It is important to stress that our estimates were not population-based but registry-based and included patients already diagnosed with iMCD; therefore, our estimates reflect the prevalence of clinical cases of iMCD rather than a population-based prevalence of the condition (see detailed explanation below under study limitations), which may also explain the lower prevalence found in our study. Additionally, previously published epidemiological studies, mainly from Asian and US sources, have reported the estimated prevalence of CD and MCD, but they lack specific data on iMCD (17, 18, 20), probably because, as registries are used, the process to assess the criteria for iMCD diagnosis was not formally implemented, unlike in our study. Therefore, the present study has the added value of reviewing each case by applying international, evidence-based consensus criteria for iMCD (7). In fact, seven out of 37 patients (19%) were excluded because they did not fulfill the iMCD diagnostic criteria.

The percentage of males (50%) and the median age at diagnosis (48 years) of the 30 patients in our study are in accordance with the data reported in the literature (19, 21, 22). Among the clinical data prevalence, 23 (77%)were classified as the iMCD-NOS subtype, whereas seven patients (23%) were classified as the iMCD-TAFRO subtype. Similarly, 9 patients (30%) presented with severe disease variants. These results are consistent with the findings of a large retrospective study conducted in China, in which 26% of the patients in the iMCD group were classified to have severe disease at the time of their diagnosis (22); furthermore, they reported a high proportion of patients with iMCD-NOS (76%), which is also in line with our study findings. Conversely, in the ACCELERATE natural history registry, 77% of patients presented with severe iMCD at diagnosis, and 60% met the iMCD-TAFRO diagnostic criteria (21). Nonetheless, ACCELERATE investigators stated that the reason for the high proportion of severe patients with a TAFRO diagnosis might be that patients with more severe disease variants were more likely to be enrolled in the registry (21).

The most frequent comorbidities that were present prior to the iMCD diagnosis recorded in the present study were autoimmune and inflammatory diseases (n = 8) and diabetes mellitus (n = 3). Similarly, hypertension (n =5) and hypothyroidism (n = 2) were the most common comorbidities diagnosed after the iMCD diagnosis. Notably, only one thrombotic event was reported in our study. These comorbid conditions are not in accordance with those reported in the ACCELERATE registry before and after disease diagnosis (21). The discrepancies between the two studies may be due to the different characteristics of their respective populations.

Histopathological analysis of excisional lymph node biopsies revealed that the plasmacytic subtype was the most prevalent (43%) in the whole cohort, which was driven mainly by the iMCD-TAFRO subgroup of patients (57%). The hypervascular and mixed subtypes accounted for 13 and 33% of the patients with iMCD, respectively. These results are consistent with those reported by Zhang et al. (22) in a study conducted in China (21) in which 52% of patients with iMCD presented with a histopathologic plasmacytic subtype. However, our study results contrast with the findings from the ACCELERATE registry, where only 7% of subjects were classified within the plasmacytic subtype (21, 23). Notably, excisional lymph node biopsies were performed in 30 patients (100%) enrolled in our study, which is in accordance with the recommendations of the International and National Consensus (7, 14), to allow a more precise and complete evaluation of CD histopathological features, including grading and exclusion of neoplastic conditions. Thus, although other lymph node biopsy techniques, such as fine needle aspiration and core needle biopsy, can be informative in the early stages of CD, the definite diagnosis of CD requires excisional lymph node biopsy (14).

Prior to the inclusion treatment patterns reported in our study, the most frequent first-line therapy was anti-IL-6 mAb with/without corticosteroids (31·8%), followed by rituximab with/without corticosteroids (22·7%) and corticosteroids in monotherapy (22·7%). Nonetheless, corticosteroids, either as monotherapy or combined therapy, were the most commonly prescribed drugs in first-line therapy (68·2%). These findings, although similar to those reported by other investigators (19), are not in accordance with published evidence-based treatment guidelines (8), which recommend anti–interleukin-6 treatment as the first-line treatment, which is the only drug approved for iMCD in Spain. In our study, the median time from the diagnosis of iMCD until inclusion in the study was 47 months, and 22 patients were diagnosed with iMCD after 2016, indicating that more than 50% of patients had an iMCD diagnosis after the International Guidelines for iMCD were published (7), and siltuximab has been approved for the treatment of iMCD since 2016 (24). The results from the current treatment are quite different, showing that in patients on treatment, siltuximab accounted for 90% of the administered therapy These results support the positioning of anti-IL-6 agents as a preferred, long-term treatment strategy in this real-world patient cohort. Importantly, at the time of inclusion in this study, the vast majority of patients had achieved a complete response, partial response, or stable disease, suggesting that most patients had a favorable prognosis, and that IL-6 blockade could provide durable disease control in routine practice; however, these latter findings should be taken with caution, because, besides other study limitations, a the cross-sectional design does not allow for establishing causality.

The major strength of our study is that the diagnosis of iMCD was confirmed by an independent scientific committee on the local pathology reports from the participating sites and after the mandatory performance of an excisional lymph node biopsy in all enrolled patients. Medical records were the main source of clinical and pathological reports submitted to the scientific committee.

This study has several limitations. As the information used for the assessment was retrospectively collected, some missing data could not be discarded. More importantly, this was not a population-based study and did not use a probability sampling method. Instead, it was based on a clinical registry using the catchment area of the corresponding medical center as the denominator. This reflects the difficulties in conducting prevalence studies on rare diseases (25). In fact, the design of previous studies evaluating the prevalence and/or incidence of CD also faced these challenges and used claim databases (17, 19) or medical records from specific medical centers (18, 20). This design limits the representativeness of the sample. Patients diagnosed with iMCD face a challenging prognosis, with mortality rates ranging from 23% to 45% within the first five years, increasing to approximately 60% over a ten-year period (26) Therefore, prevalence rates are significantly impacted by this poor prognosis, and this information should be complemented by the incidence rates to have a more complete picture of the disease burden in terms of frequency. To be included, patients had to be under follow-up at the beginning of the study in a tertiary medical center, which introduces a referral bias since patients could differ from those in the community or general population, a bias that is controlled in population-based studies. Patients, especially those with mild disease, could be managed at a small center and thus not be captured by our design. Overall, we believe that our study is an approach to study the prevalence of the disease that reflects the prevalence of clinical cases of iMCD and is likely to underestimate the population-based prevalence of this condition.

In conclusion, this study suggests that the prevalence of clinical cases of iMCD in Spain is low and comparable to that reported in previously published epidemiological studies. IL-6 blockade with siltuximab is the most commonly used therapeutic strategy in our cohort, in line with guideline recommendations. Under treatment, most patients have a favorable prognosis, with a few presenting with disease progression.

## Data Availability

The datasets generated and/or analyzed during this clinical study are not publicly available because of restrictions related to the data. Anonymized datasets may be provided by the corresponding author upon reasonable request. Requests to access the datasets should be directed to andres.gonzalez@salud.madrid.org.
