# Supplementary material for: Characteristics of idiopathic multicentric Castleman disease in Spain: results of the registry-based ARCANA multicenter study
Source: Front Oncol. 2026 Oct 19;16:1963499. doi: 10.3389/fonc.2026.1963499 (PMC13724649; doi:10.3389/fonc.2026.1963499)
Supplement: Supplementary file 1 [file DataSheet1.docx]

**Supplementary material**

**Supplementary Tables**

**Supplementary Table 1.** Screening failures

| **Patient** | **Reason** |
| --- | --- |
| 1 | Lymph node biopsy was not diagnostic for iMCD |
| 2 | It was an UCD diagnosis, confirmed by radiological assessment |
| 3 | It was a case of POEMS syndrome |
| 4 | The patient did not meet the required minor criteria of iMCD |
| 5 | The patient did not have an idiopathic diagnosis. A concomitant Hodgkin’s lymphoma interfered with the diagnosis of iMCD. |
| 6 | The patient did not have confirmation of an idiopathic diagnosis. HHV-8 negativity was not confirmed. |
| 7 | The patient did not meet the required minor criteria of iMCD |

iMCD, idiopathic multicentric Castleman disease; POEMS, polyneuropathy, organomegaly, endocrinopathy, monoclonal gammopathy, and skin changes; UCD, unicentric Castleman disease.

**Supplementary Table 2.** Therapy by line of treatment

| **Line** | **Treatment** | **N** | **(%)*** |
| --- | --- | --- | --- |
| **1** | Anti-IL-6 with/without corticosteroids | 7 | 31.8 |
|  | Rituximab with/without corticosteroids | 5 | 22.7 |
|  | Corticosteroids | 5 | 22.7 |
|  | Chemotherapy-based regimen | 4 | 18.2 |
|  | Immunomodulator with/without corticosteroids | 1 | 4.5 |
|  | **Total** | **22** | **100.0** |
| **2** | Rituximab with/without corticosteroids | 5 | 31.3 |
|  | Anti-IL-6 with/without corticosteroids | 4 | 25.0 |
|  | Chemotherapy-based regimen | 3 | 18.8 |
|  | Immunomodulator with/without corticosteroids | 2 | 12.5 |
|  | Other (radiotherapy) | 1 | 6.3 |
|  | Corticosteroids | 1 | 6.3 |
|  | **Total** | **16** | **100.0** |
| **3** | Chemotherapy-based regimen | 4 | 40.0 |
|  | Immunomodulator with/without corticosteroids | 3 | 30.0 |
|  | Rituximab with/without corticosteroids | 2 | 20.0 |
|  | Anti-IL-6 with/without corticosteroids | 1 | 10.0 |
|  | **Total** | **10** | **100.0** |
| **4** | Chemotherapy-based regimen | 2 | 33.3 |
|  | Anti-IL-6 with/without corticosteroids | 2 | 33.3 |
|  | Rituximab with/without corticosteroids | 1 | 16.7 |
|  | Immunomodulator with/without corticosteroids | 1 | 16.7 |
|  | **Total** | **6** | **100.0** |
| **5** | Chemotherapy-based regimen | 2 | 50.0 |
|  | Rituximab with/without corticosteroids | 1 | 25.0 |
|  | Anti-IL-6 with/without corticosteroids | 1 | 25.0 |
|  | **Total** | **4** | **100.0** |
| **6** | Rituximab with/without corticosteroids | 1 | 50.0 |
|  | Anti-IL-6 with/without corticosteroids | 1 | 50.0 |
|  | **Total** | **2** | **100.0** |
| **7** | Rituximab with/without corticosteroids | 2 | 100.0 |
|  | **Total** | **2** | **100.0** |

Anti-IL-6, Anti-interleukin-6

*Percentages calculated over the total number of patients in each line (line 1=22, line 2=16, line 3=10, line 4=6, line 5= 4, line 6=2, and line 7=2).

**Supplementary Figures**

**Supplementary Figure 1.** Study design
